# Longitudinal Association between Urbanicity and Total Dietary Fat Intake in Adults in Urbanizing China from 1991 to 2015: Findings from the CHNS

**DOI:** 10.3390/nu12061597

**Published:** 2020-05-29

**Authors:** Chang Su, Xiaoyun Song, Haojie Hu, Wenwen Du, Huijun Wang, Bing Zhang

**Affiliations:** 1 National Institute for Nutrition and Health, Chinese Center for Disease Control and Prevention, Beijing 100050, China; suchang@ninh.chinacdc.cn (C.S.); sxydljk@126.com (X.S.); 15732683256@163.com (H.H.); Duww@ninh.chinacdc.cn (W.D.); wanghj@ninh.chinacdc.cn (H.W.); 2 Dalian Center for Disease Control and Prevention, Dalian 116021, China

**Keywords:** urbanicity, multilevel model, fat intake, China

## Abstract

Few studies have examined the longitudinal association between urbanicity and dietary fat intake in Chinese adults. A population-based longitudinal observational study was carried out in Chinese adults aged 18–65 from the China Health and Nutrition Survey. Three consecutive 24 h dietary recalls were used to assess dietary fat intake. Multilevel models were used to explore the relationship between urbanicity and dietary fat intake. People in the highest urbanicity quartile had the increments of 7.48 g/d (95% CI:5.42–9.58) and 8.92 g/d (95% CI: 7.03–10.80) in dietary fat intake, 2.86 (95% CI: 2.29–3.44) and 2.69 (95% CI: 2.13–3.25) in proportion of energy from total fat, and odds ratios (ORs) for the risk of excess dietary fat intake of 1.84 (95% CI: 1.65–2.05) and 2.01 (95% CI: 1.78–2.26) for men and women, respectively, compared to the lowest quartile after controlling for potential confounders. These results indicate that urbanicity was an important factor influencing dietary fat intake among Chinese adults. Aggressive nutritional education action coupled with governmental guidelines and programs tailored for the Chinese population are required to promote less dietary fat intake, especially in those adults living in less urbanized areas and whose dietary fat intake is ≥ 30% of their total energy intake per day.

## 1. Introduction

Urbanicity is commonly defined as the change of urban size, density, and heterogeneity in places and migration into cities and is described as the presence of city features or the degree to which a place exhibits city characteristics [1]. In the past few decades, China has experienced the largest population migration in the world, resulting in an increase in the proportion of urban population from 21% in 1982 to 51% in 2011 [2]. A number of studies have shown that rapid urbanicity is associated with adverse health consequences such as pre-diabetes or diabetes, hypertension, high levels of total cholesterol, low density lipoprotein-cholesterol, triglycerides and C-reactive protein, as well as low levels of high density lipoprotein-cholesterol [3,4,5,6]. Meanwhile, the Chinese population has been undergoing a unique nutrition transition towards choosing diets high in energy and fat with rapid urbanicity in China [5].

In the Chinese population, a relatively low percentage of adults consumed diets with high fat intake 20 years ago, while the proportion of high fat (energy from total dietary fat ≥30% of total energy) consuming adults has been rising recently [7,8]. Some previous studies indicated that high fat intake was a major contributor to the development of being overweight and obesity and high fat intake increased the level of low density lipoprotein(LDL) [3,5,9,10,11,12]. Established by previous scientific evidence, the optimal proportion of energy from total dietary fat is considered to be lower than 30%, which is set as a recommended value for keeping healthy [8].

Previous studies in developed and developing countries showed that dietary fat intake differed between urban and rural populations, but these researches tended to focus on crude urban–rural comparisons or on specific populations from urban areas [13,14]. Meanwhile, a previous study had improved measurement of urbanity, which could draw information from the domains that characterize urban and rural places, was sensitive to changes over time, and could represent gradations on the continuum from rural to urban environments. The traditional classification of urban/rural status could miss enormous information on food markets, quantity of supermarkets, and modern eating establishments that constituted an urban environment and had a potential influence on dietary behaviors such as fat intake at different stages of urbanicity, particularly in the context of rapid social and environmental change [1,2]. So, the impact of rapid urbanicity on populations’ dietary fat intake remained less well characterized.

China provides a unique opportunity to study how urbanicity is related to dietary fat intake in populations due to unprecedented economic growth and rapid urbanicity accompanied by notable changes in lifestyle and landscapes during the past few decades. Rates of change and patterns have not been universal. A lot of Chinese rural areas have seen rapid population growth and changes within specific dimensions, such as more access to supermarkets and modern eating establishments, while some urban areas have lost walkways, cycle ways, and outdoor exercise facilities with economic development [15]. Given this heterogeneity, understanding the overall impact of urbanicity with changes of fat intake was very important for informing community-level prevention efforts. Consequently, to address these gaps, data from Chinese adults (ages 18–65 years), which has seen up to nine waves in the China Health and Nutrition Survey (CHNS), was used to examine the temporal trends in dietary fat and investigated the effect of the previously validated urbanicity scale on dietary fat intake in Chinese adults in the last two decades.

## 2. Materials and Methods

### 2.1. Study Population

Data for the present study came from the CHNS, an ongoing prospective study that was designed to provide representation of urban, rural, and suburban areas varying substantially in demographic, socioeconomic development, public resources, and health and nutritional information, with a focus on examining diet, physical activity, health, and behavior changes in the background of rapid change of urbanicity and the economy [16]. As the only large-scale longitudinal study of this kind in mainland China, the first round of the CHNS was conducted in 1989 and has been followed up every two to four years. So far, there have been a total of 10waves of surveys, conducted in 1989, 1991, 1993, 1997, 2000, 2004, 2006, 2009, 2011, and 2015. The original 1989 survey used multistage, random cluster sampling and a weighted sampling method to select the participating communities. Using this sampling strategy, participating communities were selected from two cities, one large (usually the provincial capital) and one small (usually a lower income one), and four counties (stratified by income: one high, one low, and two middle income counties) in each province. Within each city, 2 urban and 2 suburban neighborhoods were randomly selected; within each county, one community in the capital city and three rural villages were selected. The neighborhoods and villages were defined as communities in this study. In each community, 20 randomly chosen households were surveyed and all individuals living within the household were interviewed. The 2015 round included 7200 households within 360 communities (60 urban neighborhoods, 60 suburban neighborhoods, 60 towns, and 180 villages) across 15 diverse provinces (Heilongjiang, Liaoning, Shandong, Henan, Hubei, Hunan, Jiangsu, Guizhou, Guangxi, Shanxi, Yunnan, Zhejiang, Chongqing, Shanghai, and Beijing). Additional details regarding the CHNS are provided elsewhere [17].

**Figure 1:**
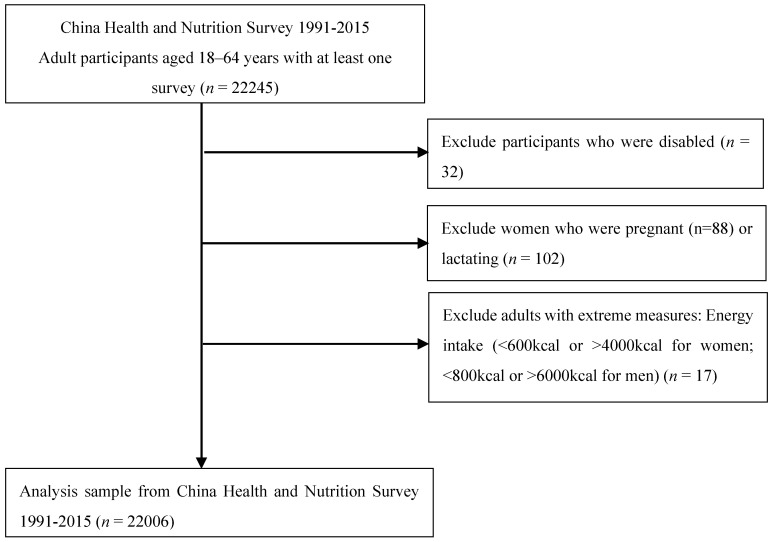
Flow chart of the 1991–2015 China Health and Nutrition Survey(CHNS) analysis sample.

Our sample included nine rounds of survey data collected in 1991, 1993, 1997, 2000, 2004, 2006, 2009, 2011, and 2015. All the adults (aged 18–65) with complete data on socioeconomic status, demographics, and three-day 24 h dietary recalls in a survey year were considered eligible subjects. We did not use data from the 1989 wave because only young adults aged 20–45 years old were included. We excluded participants who was currently pregnant or lactating (*n* = 88) or disabled (*n* = 32) at the time of data collection. Additionally, participants who reported extreme energy intakes (<600 kcal or >4000 kcal for women; <800 kcal or >6000 kcal for men) (*n* = 17) were considered as having invalid dietary data and were excluded from the analysis (Figure 1) [18]. Our final analysis sample consisted of 22,006 participants (10,768 males and 11,238 females) clustered in 360 communities, resulting in 77,351 total observations across the nine survey years. All procedures involving human subjects were approved by the National Institute for Nutrition and Health, the Chinese Center for Disease Control and Prevention (No.201524-1), and the Institutional Review Committees of the University of North Carolina at Chapel Hill and written informed consent was obtained from all the CHNS participants.

### 2.2. Dietary Data

In the CHNS, dietary assessment was based on a combination of three consecutive 24 h recalls at the individual level and a food inventory at the household level taken over the same three-day period.

Individual dietary intake data were collected by asking every household member to report all of the food which they had consumed away from home and at home over the previous 24 h. Using food models and picture aids, well trained field interviewers recorded the amounts and types of food consumed, types of meal consumed, and places of consumption for all food items during the previous day. The three consecutive days were randomly allocated to start from Monday to Sunday and were almost equally balanced across the 7 d of the week for each sampling unit. Household food consumption was determined by the weighing and measuring technique to examine changes in the inventory from the beginning to the end of the survey. All foods and condiments remaining after the last meal before initiation of the survey were weighed and recorded. During the three days of the survey, all home production foods and purchases from markets were also weighed and recorded. Preparation waste was estimated when weighing was not possible. At the end of the survey, all the remaining foods were again weighed and recorded. The food scale (TANITA KD-321) was calibrated before use and the minimum scale range of the food scale was 0.1 g. The amount of food in each dish was estimated from the household inventory and the proportion of each dish consumed was reported by each person that was interviewed [19].

The data quality control was ensured in two ways. Firstly, all field interviewers were taken through a high-standard training course for at least three days in the collection of dietary data in each survey [19,20,21]. Moreover, the quality control staffs checked the quantity of the data collection and compared the individual’s dietary intake calculated from the 24 h recall data with an individual’s average daily dietary intake based on the household inventory. Where there were significant discrepancies in estimated intakes, the field interviewers revisited the households and individual’s questions and inquired about the individual food consumption again.

The calculation of each individual’s mean daily fat intake (g) values for each food item were obtained from the dietary intake data collected in the CHNS, which were derived from the Chinese Food Composition Table. The 1991 Food Composition Table was used for the survey of 2000 and previous years. A newer version of the Food Composition Table (2002 and 2004) was used for the 2004 and 2006 surveys and the latest version (2009) was used for the 2009, 2011, and 2015 surveys [22]. The excess of dietary fat intake was defined as a proportion of energy from total fat ≥30% for men and women [8].

### 2.3. Urbanicity Scale

Urbanicity was defined using a component index that used community measures of infrastructure, services, and population to capture major dimensions of what is termed urbanicity. Urbanicity, which is designed and validated for the CHNS, has high validity, reliability, and temporal stability. The measure of urbanicity was according to our previous study [23,24] and another study [25]. In the present study, the detailed individual-level and community-level data were used to create a multi-component scale defined by using a 12-component index including physical, social, cultural, and economic environment. According to the study of Jones Smith and Popkin [1], scoring distributions were variable across components, so the median value of a component value was set as half the total points and each of the components were scaled to 0–10. Points were allocated on the basis of presence of infrastructure or facilities in each of 12 domains (1) population density (i.e., population per km^2^); (2) types of economic activity (i.e., daily wage for an average male worker and % community engaged in nonagricultural labor); (3) traditional market (i.e., types, distances, and business hours of food and fuel markets); (4) modern markets (i.e., quantity of supermarkets and modern eating establishments); (5) transportation and infrastructure (i.e., road types and availability of transit services); (6) sanitation (i.e., availability of treated water and presence of excrement in public space); (7) communications (i.e., media availability in the community and percent of households with electronics); (8) housing (i.e., availability of electricity, water, gas, flush toilet); (9) education (i.e., average highest attained level for adults); (10)diversity (i.e., variation in community education level and income level); (11) health infrastructure (i.e., type, distance, and number of health services in the community); and (12) social services (i.e., availability of insurance and child care centers), with a range of 0–120 (with a higher score reflecting more urban characteristics across multiple domains). This scale represents a broad-based measure of the elements of modernization that have potential health effects. This scale has been validated for content validity, reliability (a = 0.85–0.89 across all study years), and stability across study years (r = 0.90–0.94) [1].

In the present study, the urbanicity index was calculated from the total scores of the 12 domains and these indexes were then categorized into quartile (Q1, Q2, Q3, and Q4) by gender to reflect the degree of urbanicity for each community. In addition, we examined whether total urbanicity score was associated with the likelihood of fat intake and fat from total energy in both men and women. Furthermore, we explored how the urbancity level related to the risk of high fat intake since China’s rapid urbanization has been characterized by considerable spatial and temporal variability in environmental and social changes. The lowest quartile of score of urbanicity was the reference group.

### 2.4. Assessment of Other Covariates

Well trained interviewers used standard questionnaires to collect detailed information on annual household income, individual education level, and physical activity. We used the formula of annual family income/household size to calculate per capita annual income of household. The per capita annual income of the family in each survey was inflated to that of 2015 by adjusting for the consumer price index (CPI) and was then divided into tertiles (low-, medium-, and high-level). An individual’s education level was based on the number of years of formal education completed in regular school and was then classified into three categories including primary school or less (≤6 years), middle school (>6 years and ≤9 years), or high school or college (>9 years). A detailed 7-day physical activity recall instrument was used to report the respondents’ time per week spent in four domains of physical activity, including occupational, domestic, active leisure, and travel physical activity, and all activities were assigned metabolic equivalent of task (MET) values using the Compendium of Physical Activities [26,27]. We used the MET hours per week that account for both the average intensity of each activity and the time spent in each activity to show the sum amount of physical activity of each respondent.

The other covariates that could influence the association between urbanicity and fat intake, gender (male or female), age (in years), alcohol consumption (drinking or no drinking), and smoking status (a daily smoker, not a daily smoker), were included in the analysis.

### 2.5. Statistical Analysis

We categorized participants into quartiles of urbanicity index and tested for differences in individual-level variables across the quartiles of urbanicity for men and women at baseline using one-way analysis of variance (ANOVA) or analysis of covariance (ANCOVA) for continuous variables and chi-square tests for categorical variables.

A multilevel mixed-effects linear regression model was constructed to estimate fat intake and energy from fat in relation to level of urbanicity and a multilevel mixed-effects logistic regression model was performed to assess the risk of the excess of dietary fat intake after adjusting for covariates including survey year, age, individual income, educational level, smoking status, alcohol consumption, physical activity and total energy intake, and baseline dietary fat intake. We calculated regression coefficients (95% confidence intervals [CIs]) and odds ratios (ORs) (95% CIs), respectively. We used the likelihood ratio tests to examine the effect modifier in initial model specification and observed a significant interaction between gender and fat intake. Therefore, all models in the present study were subsequently stratified by gender. In addition, we evaluated linear trends by assigning participants the median value for quartiles of urbanicity index and we entered this variable as a continuous term in the regression model. All statistical tests were two-tailed and *p* < 0.05 was regarded as significantly different. All analyses were conducted using SAS (version 9.2 SAS Institute, Cary, CA, USA) and Stata version 13.0 (College Park, TX, USA).

## 3. Results

**Figure 2:**
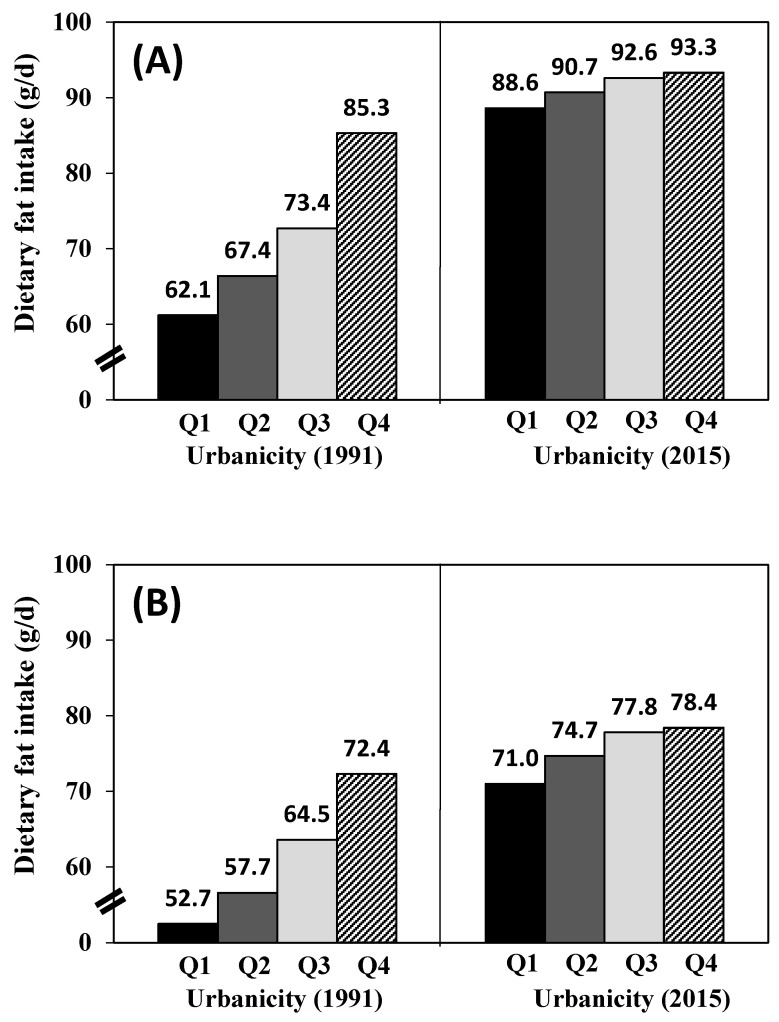
Dietary fat intake with quartile of urbanicity in 1991 and 2015: (**A**) men; (**B**) women.

**Figure 3:**
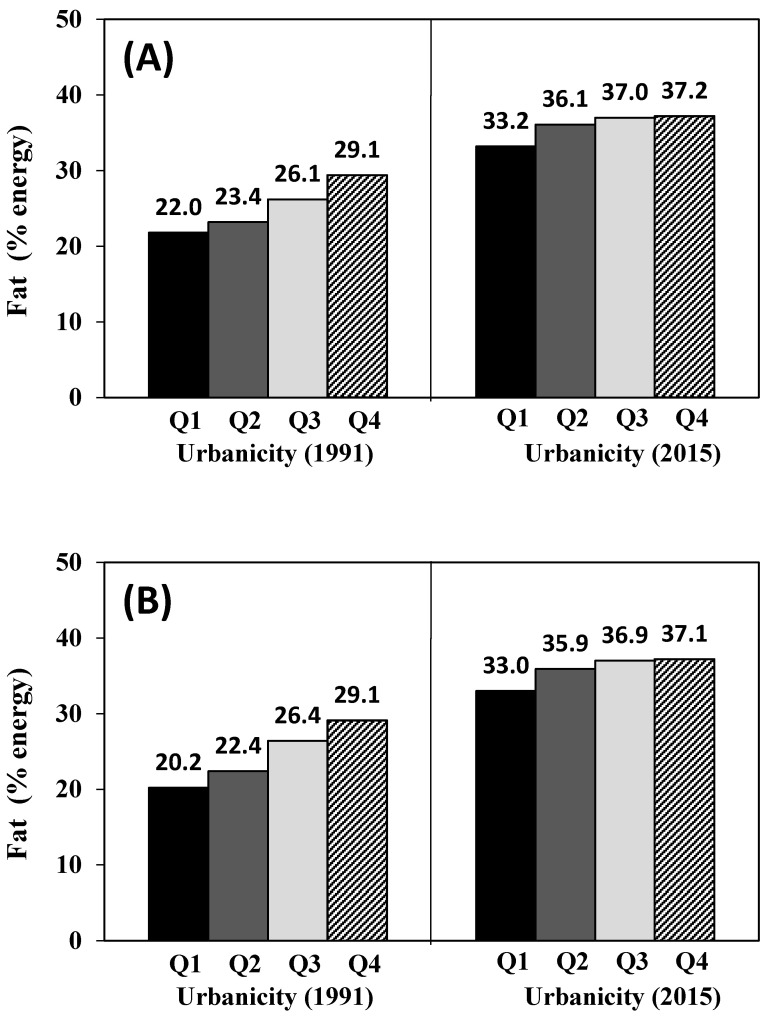
Energy of fat in total energy intake with quartile of urbanicity in 1991 and 2015: (**A**) men; (**B**) women.

**Figure 4:**
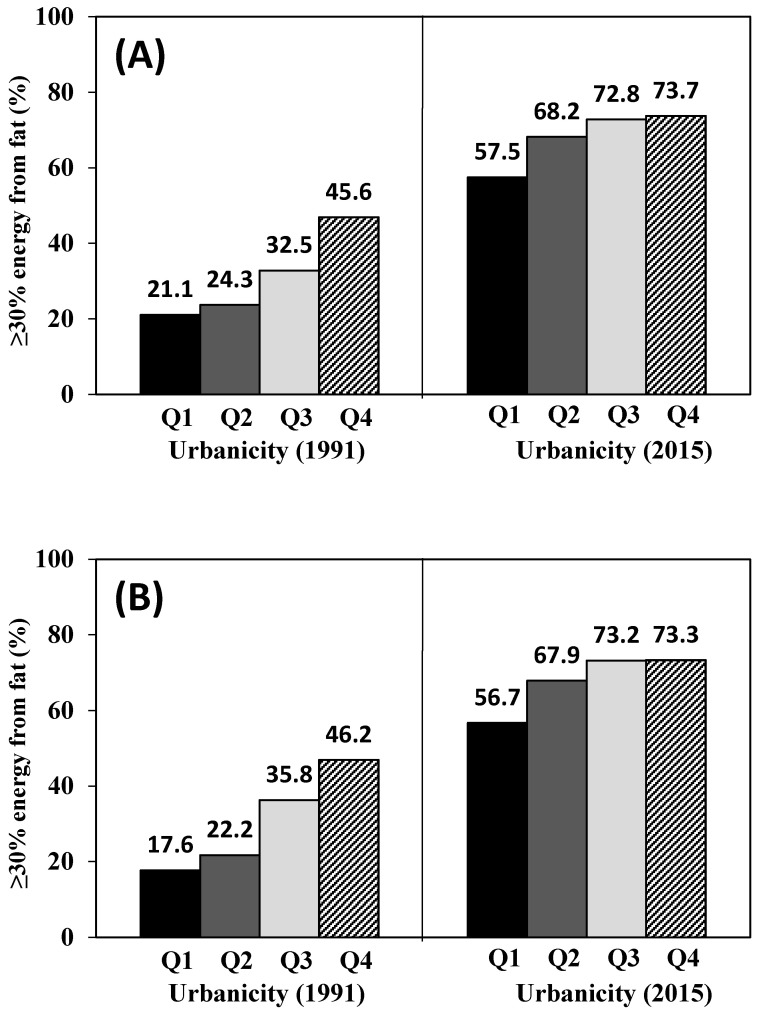
≥30% energy from fat (%) with quartile of urbanicity in 1991 and 2015: (**A**) men; (**B**) women.

Table 1 presents the baseline characteristics of participants for each gender by quartiles of urbanicity index. The average urbanicity index score from the lowest to the highest quartile ranged from 26.5 to 67.4 in male and from 26.4 to 67.3 in female. Compared to those in the lower urbanicity quartiles, participants in the higher quartiles tended to be older, have higher incomes and educational levels, and do less MET-h/week of physical activity in both men and women. The percentage of smokers in the male subjects in the highest urbanicity quartile was less than that in the male subjects in the lowest quartile. However, the percentage of alcohol drinkers in female subjects in the highest quartile was higher than that in female subjects in the lowest quartile. Figure 2, Figure 3 and Figure 4 show that higher urbanicity level was associated with higher dietary fat intake, proportion of energy from total fat (% of energy), and the percentage of population with ≥30% energy from fat in both 1991 and 2015. In addition, it was notable that during the past 25 years (from 1991 to 2015), fat intake in the lowest urbanicity quartile increased by 26.5 g/d for males and 18.3 g/d for females, while fat intake in the highest urbanicity quartile increased by 8.0 g/d for males and 6.0 g/d for females.

Table 2 shows the results of a longitudinal association between urbanicity level and fat intake and proportion of energy from total fat (% of energy) stratified by gender. The net effect of the decline from the highest quartile of urbanicity to the lowest quartile of urbanicity was a significant decrease of 7.5 g/d (β: 7.48, 95% CI: 5.42–9.58) and 6.4 g/d (β: 6.35, 95% CI: 4.65–8.05) in dietary fat intake in men and women, respectively. The increase in urbanicity from the lowest quartile to the highest-quartile was linked with a significant increment of 2.9 (β: 2.86, 95% CI: 2.29–3.44) and 2.7 (β: 2.69, 95% CI: 2.13–3.25) in the proportion of energy from total fat in men and women, respectively.

Table 3 summarized odds ratios of a multi-level mixed-effects logistic regression model used to examine the effects of urbanicity level on the odds of excessive dietary fat intake. A significant association between urbanicity level and the odds of excessive dietary fat intake was observed in both men and women. Men who lived in the most urbanized areas were 1.84 times (95% CI: 1.65–2.05) more likely to have excessive dietary fat intake than those who lived in the lowest urbanized areas. Similarly, the odds of excessive dietary fat intake in women who lived in the most urbanized areas were 2.01 times (95% CI: 1.78–2.26) higher than that of women who lived in the lowest urbanized areas after additional adjustment for baseline dietary fat intake.

## 4. Discussion

Like previous studies comparing rural and urban differences in fat intake in some undeveloped regions or developing countries such as China, our results showed that adults living in more urbanized areas were more likely to have more fat intake than those living in less urbanized areas. However, unlike most previous studies, which focused on a single geographically or governmentally defined dichotomous measure of rural/urban location, we used a multi-component measure that included information of social and physical environmental characteristics of communities that accompanied sustainable urbanicity. Given the rapidity and non-uniformity of urbanicity, the measure was particularly advantageous not only in that it allowed for a more meaningful classification of different communities rather than the traditional rural–urban dichotomy, but also the time-varying quality of the index allowed us to capture the continuum changes in urbanicity over time.

As in most countries, urbanicity in China is a critical impetus of economic development as well as an indication of socio-economic and civilization progress [28]. With the economic growth in China, the urban population will increase in the future. World urbanicity prospects shows that the urbanicity rate will reach 77.3% in 2050 in China [29]. Degree of urbanicity is a reflective indicator of community change as well as economic development in developing countries. Urbanicity has provided many benefits in living, transportation, health service, and other facilitative community aspects. However, rapid urbanicity has had certain impacts on the eating habits and overall health of individuals within developing communities, causing a significant nutrition transition in developing countries, such as Africa, China, and India [30]. Therefore, it is necessary to gain a deeper understanding of urbanicity and its role in dietary changes, especially in a way that relates to chronic disease risks. Although several studies have explored relationships between urbanicity and dietary fat intake, these studies did not utilize system analysis and long term observations [25,31,32,33]. The urbanicity index in the present study is a comprehensive indicator of urbanicity, which includes 12 defined characteristics of an urbanized community, so it may have the potential to demonstrate an overall community change and serve as a better determinant of dietary behavior change. To the best of our knowledge, the present study is the first to evaluate the longitudinal association of urbanicity level with dietary fat intake in Chinese adults with an emphasis on the odds of excess of dietary fat intake.

The urbanicity level had a dramatic impact on individual’s income, education, and behavior habits in the present study. We found that the individuals living in communities of a higher urbanicity level had higher income and educational levels in both males and females and the proportion of smokers decreased in males but increased in females when the urbanicity level increased, which was consistent with a previous study [34]. Studies have shown that increasing urbanicity has predominantly negative consequences on health, for example, leading to increased body mass index, low physical activity, and reduced fruit and vegetable consumption [35]. High urbanicity level is a risk factor for cardiovascular-related chronic diseases in low- and middle-income countries, perhaps due to lower physical activity and unreasonable dietary patterns [36]. Our present results were in line with the above conclusion, which demonstrated that individuals from communities of the highest urbanicity level had the lowest observed physical activity. This result indicated negative associations between physical activities and urbanicity. As China continues to urbanize, efforts to increase leisure physical will be particularly critical for individuals living in areas with a higher urbanicity index.

In this population-based prospective cohort study of Chinese adults in the context of China sustainable urbanicity, we found that adults living in less urbanized communities had a lower amount of dietary fat intake and a lower percentage of population with high fat intake, while a greater amount of dietary fat intake and a higher percentage of population with high fat intake were found in communities with a higher level of urbanicity. Therefore, urbanicity level was positively associated with dietary fat intake and proportion of energy from total fat (% of energy) among Chinese adults aged 18–65. Results also showed significant associations between the percentage of population with ≥30% energy from fat and odds of the excess of dietary fat intake and urbanicity level in both males and females. The dietary fat intake of Chinese adults in each urbanicity quartile increased dramatically from 1991 to 2015. The increases were 42.5% (Q1), 34.6% (Q2), 26.2% (Q3), and 9.4% (Q4) in men and 34.7% (Q1), 29.5% (Q2) 20.6% (Q3), and 8.3% (Q4) in women. Similar trends were also found in proportion of energy from total fat (% of energy) and the percentage of population with ≥30% energy from fat. Although the present study showed a significant increase in fat intake following urbanicity from 1991 to 2015 in China, changes differed depending on different levels of urbanicity, which was consistent with other countries [37,38,39,40]. In 2015, notable increases of fat intake were seen in adults in all urbanicity quartiles. More than half of adults in the lowest urbanicity quartile consumed high amounts of fat and three-quarters of adults in the highest urbanicity quartile had a high fat intake, which increased the risk of obesity and chronic diseases. The percentage of population with ≥30% energy from fat in the lowest urbanicity quartile increased about 2.7 times (male) and 3.2 times (female) in 2015 compared to those in 1991.The differences of fat intake by level of urbanicity (Q4vsQ1) narrowed over time, with those in the lowest urbanicity quartile having experienced the largest increase in dietary fat intake during the past 25 years (23.2 g/d in 1991 and 4.7 g/d in 2015 for male; 19.7 g/d in 1991 and 7.4 g/d in 2015 for female). From our results, focusing on individuals living in less urbanized areas may be a critical area for nutritional intervention as it may have better effectiveness in the prevention and control of chronic diseases.

Comparing communities of the highest with the lowest quartile of urbanicity, ORs (95% CI) of having excessive dietary fat intake in males and females were 1.84 (1.65, 2.05) and 2.01 (1.78, 2.26) after controlling for individual factors including surveyed year, age, gender, income, educational level, smoking, and alcohol consumption. This provided evidence that the urbanicity level of community was an important factor in determining dietary fat intake in developing countries. However, in developed countries, dietary fat intake is more strongly associated with individual lifestyle factors than characteristics of the community [41]. Therefore, based on different levels of urbanicity, there is a great difference in the patterns of dietary fat intake between developing and developed countries, and the role of community factors in changing dietary fat intakes may have a lesser effect when the degree of urbanicity reaches a high level [42,43,44,45].

The present study has some strengths. The sample size was large with a wide age range. The quality of data was guaranteed by staff trained in the study’s methodology and the simultaneous standardization of different parameters by the same scientists. The use of the individual-level 24 h recalls taken on three consecutive days’ recall method could improve the accuracy of dietary recalls and hence, the analysis and results [46]. Moreover, mixed-effect modeling could reduce bias and increase the accuracy of estimates.

Some possible limitations warrant cautious interpretations of our results. First, the complex sampling design we used facilitated our sampling execution, which inevitably leads to the loss of sampling accuracy. In this study, we tried to make up for the loss of sampling accuracy by enlarging the sample size. Second, due to some supplements and changes within households, there were losses to follow up, which could potentially reduce the efficacy in the analysis as well as increase confounding factors [16]. In addition, this study did not look at the trends and patterns of saturated, monounsaturated, and polyunsaturated fatty acid intake as well as n-3, n-6, and n-9 fatty acids for lack of relating information in the Food Composition Tables of China. Besides, the effects of traditional and local culture on dietary fat intake were not discussed in this study, although it has been discussed in other previous studies [47], however the effects are currently unclear. Another consideration is that populations with different birth years might show differences in fat intake change, so this could provide more evidence of other factors having a greater impact on changes in dietary fat intake using different birth cohorts for further study.

## 5. Conclusions

Findings from the present study indicated that the dominant dietary pattern among Chinese adults has shifted toward a high fat diet, which implies that China had experienced a rapid nutrition transition following rapid urbanicity during the past two decades. It was worth noting that the largest transition in dietary fat intake occurred in Chinese adults living in less urbanized communities. Given the positive link between excess dietary fat and unhealthy consequences, our study results suggest that the disease burdens caused by the nutrition transition might shift to residents in communities of a low urbanicity level in China in the future. Aggressive nutritional education action coupled with governmental guidelines and programs tailored for the Chinese population are required to ensure a more balanced diet for adults in China’s rapid urbanicity process.

## Figures and Tables

**Table 1:** Baseline descriptive characteristics of participants for each gender by quartiles of community-level urbanicity, CHNS 1991 ^a^.

	Men				Women			
	Q1 ^b^ (*n* = 988)	Q2 (*n* = 979)	Q3 (*n* = 974)	Q4 (*n* = 977)	Q1 (*n* = 1076)	Q2 (*n* = 1091)	Q3 (*n* = 1098)	Q4 (*n* = 1066)
Age (years) ^c^	38.6 (0.4)	37.7 (0.4)	38.0 (0.4)	39.9 (0.4) **	37.5 (0.4)	36.5 (0.4)	37.6 (0.4)	39.2 (0.4) ***
Income tertiles (%)								
Low	54.2	36.9	26.5	11.7 ***	52.9	35.7	29.5	10.3 ***
Middle	29.4	32.1	34.3	38.6 ***	28.6	34.1	33.6	38.7 ***
High	16.4	31.0	39.2	49.8 ***	18.5	30.2	36.9	51.0 ***
Education (%)								
None/primary	62.3	50.5	43.3	27.9 ***	80.4	68.6	55.2	37.4 ***
Middle school	28.3	35.5	36.4	33.4 ***	16.6	24.8	30.0	31.4 ***
High school+	9.4	14.0	20.3	38.7 ***	3.0	6.6	14.8	31.2 ***
Current smoker (%)	66.8	64.7	60.8	62.5 *	2.4	3.5	5.4	4.0 **
Alcohol consumption (%)	59.6	61.0	59.3	62.6	10.6	11.8	12.4	13.5 *
Total energy intake (kcal/day)	2645.0 (23.6)	2628.5 (24.0)	2539.6 (24.0)	2603.3 (24.0) *	2444.2 (18.8)	2331.0 (18.7)	2188.1 (18.7)	2199.4 (18.9) ***
Dietary fat intake (g/day)	62.1 (1.2)	67.4 (1.2)	73.4 (1.2)	85.3 (1.2) ***	52.7 (1.0)	57.7 (1.0)	64.5 (1.0)	72.4 (1.0) ***
Fat (% energy)	22.0 (0.4)	23.4 (0.4)	26.1 (0.4)	29.1 (0.4) ***	20.2 (0.3)	22.4 (0.3)	26.4 (0.3)	29.1(0.3) ***
≥30% energy from fat (%)	21.2	24.3	32.5	45.6 ***	17.6	22.2	35.8	46.2 ***
Physical activity (MET hours/week)	528.6 (6.7)	474.4 (6.8)	321.6 (6.8)	182.2 (6.8) ***	629.0 (7.2)	558.8 (7.1)	375.5 (7.1)	210.7 (7.2) ***
Urbanicity index(score)	26.5 (0.1)	39.6 (0.1)	54.3 (0.1)	67.4 (0.1) ***	26.4 (0.1)	39.6 (0.1)	54.1 (0.1)	67.3 (0.1) ***

^a^ ANOVA or ANCOVA tests were used for continuous variablesand chi-square tests were used for categorical variables across quartiles of urbanicity within gender. Adjusted by age for total energy intake, dietary fat intake, percentage of energy from fat, and physical activity. ^b^ Q = quartile; MET = metabolic equivalent of task. ^c^ Values are expressed as means with standard error (continuous variable) or percentage (categorical variable).* *p* < 0.05; ** *p* < 0.01; *** *p* < 0.001.

**Table 2:** Regression coefficients (95% CI) of fat intake and fat (% energy) according to level of urbanicity among Chinese adults, CHNS (1991–2015) ^a^.

	Urbanicity Quartile				P-Trend ^d^
	Q1 ^b^	Q2	Q3	Q4	
Men					
Fat intake					
Model 1 ^e^	0.00 (ref) ^c^	3.79 (2.34, 5.24) ***	6.64 (4.67, 8.59) ***	11.38 (9.11, 13.64) ***	<0.001
Model 2 ^f^	0.00 (ref)	3.44 (4.67, 8.59) ***	6.02 (4.06, 7.98) ***	10.20 (7.91, 12.48) ***	<0.001
Model 3 ^g^	0.00 (ref)	2.62 (1.25, 3.99) ***	4.61 (2.82, 6.41) ***	7.48 (5.42, 9.58) ***	<0.001
Fat (% energy)					
Model 1	0.00 (ref)	1.16 (0.78, 1.55) ***	2.70(2.17, 3.24) ***	3.89 (3.26, 4.52) ***	<0.001
Model 2	0.00 (ref)	1.07 (0.69, 1.47) ***	2.49 (1.96, 3.03) ***	3.52 (2.89, 4.16) ***	<0.001
Model 3	0.00 (ref)	0.93 (0.57, 1.30) ***	2.19 (1.70, 2.68) ***	2.86 (2.29, 3.44) ***	<0.001
Women					
Fat intake					
Model 1	0.00 (ref)	3.31 (2.08, 4.55) ***	6.36 (4.73, 7.99) ***	8.92 (7.03, 10.80) ***	<0.001
Model 2	0.00 (ref)	2.99 (1.74, 4.23) ***	5.75 (4.11, 7.40) ***	8.17 (6.25, 10.08) ***	<0.001
Model 3	0.00 (ref)	2.27 (1.11, 3.43) ***	4.82 (3.33, 6.30) ***	6.35 (4.65, 8.05) ***	<0.001
Fat (% energy)					
Model 1	0.00 (ref)	1.25 (0.87, 1.64) ***	2.77(2.24, 3.29) ***	3.74 (3.12, 4.36) ***	<0.001
Model 2	0.00 (ref)	1.13 (0.75, 1.52) ***	2.51 (1.98, 3.04) ***	3.36 (2.74, 3.98) ***	<0.001
Model 3	0.00 (ref)	0.93 (0.57, 1.30) ***	2.17 (1.69, 2.65) ***	2.69 (2.13, 3.25) ***	<0.001

^a^ All of the models were constructed using three-level mixed-effects linear regression with maximum likelihood estimation methods. ^b^ Q = quartile. ^c^ ref = reference group. ^d^ P-trend was calculated across the quartiles of urbanicity index and the median value for each quartile was entered as a continuous term in the regression models. ^e^ Model 1 adjusted for surveyed year only. ^f^ Model 2 additionally adjusted for age. ^g^ Model 3 further adjusted for individual income, education level, physical activity, smoking status, and alcohol consumption. * *p* < 0.05, ** *p* < 0.01, *** *p* < 0.001.

**Table 3:** Odds ratio (OR) (95% CI) of the excess of dietary fat intake (energy from fat ≥30%) according to level of urbanicity among Chinese adults, CHNS (1991–2015) ^a^.

	Urbanicity Quartile				P-Trend ^d^
	Q1 ^b^	Q2	Q3	Q4	
Men					
High energy from fat (≥30%)					
Model 1 ^e^	1.00 (ref) ^c^	1.45 (1.34, 1.57) ***	2.03 (1.85, 2.23) ***	2.49 (2.22, 2.79) ***	<0.001
Model 2 ^f^	1.00 (ref)	1.40 (1.28, 1.52) ***	1.82 (1.63, 2.03) ***	2.17 (1.91, 2.47) ***	<0.001
Model 3 ^g^	1.00 (ref)	1.31 (1.19, 1.43) ***	1.68 (1.53, 1.85) ***	1.84 (1.65, 2.05) ***	<0.001
Women					
High energy from fat (≥30%)					
Model 1	1.00 (ref)	1.48 (1.36, 1.60) ***	2.35 (2.13, 2.59) ***	2.88 (2.59, 3.20) ***	<0.001
Model 2	1.00 (ref)	1.43 (1.32, 1.56) ***	2.04 (1.84, 2.27) ***	2.31 (2.06, 2.59) ***	<0.001
Model 3	1.00 (ref)	1.38 (1.27, 1.52) ***	1.79 (1.60, 2.00) ***	2.01 (1.78, 2.26) ***	<0.001

^a^ All of the models were constructed using three-level mixed-effects logistic regression. ^b^ Q = quartile. ^c^ ref = reference group. ^d^ P-trend was calculated across the quartiles of urbanicity index and the median value for each quartile was entered as a continuous term in the regression models. ^e^ Model 1 adjusted for surveyed year and age only. ^f^ Model 2 additionally adjusted for individual income, education level, physical activity, smoking status, and alcohol consumption. ^g^ Model 3 further adjusted for baseline dietary fat intake. * *p* < 0.05, ** *p* < 0.01, *** *p* < 0.001.
